# Implementation of the Robson classification in clinical practice:Lithuania’s experience

**DOI:** 10.1186/s12884-017-1625-9

**Published:** 2017-12-20

**Authors:** Justina Kacerauskiene, Egle Bartuseviciene, Dalia Regina Railaite, Meile Minkauskiene, Arnoldas Bartusevicius, Mindaugas Kliucinskas, Renata Simoliuniene, Ruta J. Nadisauskiene

**Affiliations:** Lithuanian University of Health Sciences, Eiveniu str. 2, 50167 Kaunas, Lithuania

**Keywords:** Cesarean section, Rate, Robson classification, Audit, Intervention

## Abstract

**Background:**

To determine the cesarean section (CS) rate in Lithuania, identify the groups of women that influence it using the Robson classification and to determine the impact of implementing the use of the Robson classification on the CS rate.

**Methods:**

The Robson classification was introduced in Lithuanian hospitals prospectively classifying all the deliveries in 2012. The overall CS rate, sizes of the Robson groups of women, CS rate in each group and contribution to the overall CS rate from each group was calculated and the results were discussed. The analysis was repeated in 2014 and the data were compared using MS EXCEL and SPSS 23.0.

**Results:**

Nineteen Lithuanian hospitals participated in the study. They represented 84.1% of the deliveries (23,742 out of 28,230) in 2012 and 88.5% of the deliveries (24,653 out of 27,872) in 2014. The CS rate decreased from 26.9% (6379/23,742) in 2012 to 22.7% (5605/24,653) in 2014 (*p* < 0.001). The greatest contributions to the overall CS rate were made by groups 1, 2 and 5. The greatest decrease in the CS rate was detected in group 2. The absolute contribution to the overall CS rate decreased from 4.9% to 3.8%.

**Conclusion:**

The Robson classification can work as an audit tool to identify the groups that have the greatest impact on the CS rate. It also helps to develop a strategy focussing on the reduction of the CS rate.

## Background

Cesarean section (CS) was introduced to obstetrical practice as a lifesaving procedure both for mother and her child. Paradoxically, despite the increasing CS rate, there is no further improvement in the perinatal mortality rates. On the contrary, there is a growing body of literature claiming that excessive CSs increase the risk not only of maternal and infant morbidity but also of maternal and neonatal mortality [1–3]. Therefore, focussing on strategies to avoid unnecessary CSs need to be developed.

In order to identify the most significant groups of women contributing to the growing CS rate, a universal classification of all deliveries should be implemented. Some authors claim that simply classification of deliveries itself may act as a trigger and can reduce the CS rate [4–6]. It gives an opportunity to evaluate the prevalence of CSs among various groups of women, to compare data between institutions, learn from each other and to create strategies for better results. Based on the available knowledge, the Robson classification (the Ten-group classification system) meets the current needs the best [7, 8].

The aim of our study was to implement the Robson classification (Table 1) in all Lithuanian hospitals providing obstetrical care, to identify the target groups of women who influence the CS rate the most and to determine if the implemented classification system might work as an audit tool to reduce the CS rate.

**Table 1:** The Ten-group classification system

Robson group	Description of the group
1	Nulliparous women, single cephalic, >=37 weeks, in spontaneous labour
2	Nulliparous women, single cephalic, >=37 weeks, induced or CS before labour
3	Multiparous women (excluding prev. CS), single cephalic, >=37 weeks, in spontaneous labour
4	Multiparous women (excluding prev. CS), single cephalic, >=37 weeks, induced or CS before a labour
5	Previous CS, single cephalic, >=37 weeks
6	All nullipara breeches
7	All multipara breeches (including prev. CS)
8	All multiple pregnancies (including prev. CS)
9	All abnormal lies (including prev. CS)
10	All single cephalic, <=36 weeks (including prev. CS)

## Methods

### Study setting and design

A national interventional study was performed from January 1 to December 31 of 2012 (the first study period) at the Lithuanian hospitals providing obstetrical care. It was repeated from January 1 to December 31 in 2014 (the second study period). Before the study, the Robson classification was known by the Lithuanian obstetricians and gynecologists but it had not been formally endorsed nationally and wasn’t used in everyday practice. The increasing national CS rate encouraged the Lithuanian Society of Obstetricians and Gynecologists (LSOG) to organise a special meeting where obstetricians and gynecologists and the authorities of the Lithuanian hospitals were invited. The meeting was held in December 2011. The Cesarean Section Working Group of the LSOG reminded the participants of the principles of the Robson classification at the meeting, encouraged them to classify the deliveries routinely and invited them to join the study by sending relevant data to the study coordinators. There are 33 hospitals with labour and delivery units in Lithuania. Overall, 23 and 21 hospitals in 2012 and 2014 respectively, agreed to participate in the study and reported their data. Nineteen hospitals participated in both years, while the remaining participated either in 2012 or in 2014. The reasons to withdraw participation in the study were that staff found the classification too complicated and were not interested in carrying out extra work. On the other hand the vast majority of hospitals found the study helpful and were keen to continue to take part in the study. All pregnant women admitted to a labour and delivery ward were included in the study and were classified according to the Robson classification. Any deliveries with a gestational age less than 22 weeks or with a newborn weight under 500 g were excluded from the study. The study did not require any direct contact with a patient and it could not cause any harm to them.

During the first period, all the deliveries at a certain hospital were classified by its clinicians into one of the ten classification groups. If any questions or difficulties in classifying a woman arose, two investigators (D.R.R. and E.B.) provided continuous educational assistance not only before but also during the study (in person, by e-mail or phone calls). Then summary data were sent on a monthly basis by e-mail or fax to the study investigators. In 2013 all the data from hospitals were analysed and a summit conference was organised specifically for this project. The attendees of the meeting included administrators of the participating hospitals, members of the LSOG, the Lithuanian Health Ministry and the Lithuanian Parliament. During the conference the CS rates among different hospitals and different groups of women were compared and discussed. The general consensus to try and reduce the CS rate was accepted. Moreover, it was decided that the Robson classification was clear, easy to use and a clinically valuable system which made it possible to identify the different groups of women contributing to the overall CS rate. During the second period, the classification of deliveries according to the Robson classification was repeated and the data were sent to the study investigators.

### Statistical analysis

Data were analysed using MS EXCEL and IBM SPSS Statistics 23.0 for Windows. The classified deliveries in 2012 and 2014 were compared. The z-test was used to compare equality of probabilities. Minimum sample size of 1197 subjects per group needed to detect a 5% change in the CS rate with a power of 80% and a significance level of 5%.

## Results

A total of 84.1% (23,742/28,230) of all hospital births in Lithuania in 2012 and 88.5% (24,653/27,872) of all hospital births in Lithuania in 2014 were analysed. The overall CS rate decreased from 26.9% (6379/23,742) in 2012 to 22.7% (5605/24,653) in 2014 (Table 2). This decrease was statistically significant (*p* < 0.001) (Table 2).

**Table 2:** The number of CS in each group and their contribution to the overall CS rate in 2012 and 2014

Overall cesarean section (CS) rate (%) 6379/23,742 (26.9%) in 2012 and 5605/24,653 (22.7%) in 2014										
	2012	2014	2012	2014	2012	2014		2012	2014	
Group	Number of CS over total number of women in each group		Relative size of groups (%)		CS rate in each group (%)		*p* value	Absolute contribution to overall CS rate (%)		p value
1	1289/8057	1209/8646	33.9	35.1	16.0	14.0	p < 0.001	5.4	4.9	*p* = 0.009
			8057/23,742	8646/24,653	1289/8057	1209/8646		1289/23,742	1209/24,653	
2	1158/2217	938/2239	9.3	9.1	52.2	41.9	p < 0.001	4.9	3.8	p < 0.001
			2217/23,742	2239/24,653	1158/2217	938/2239		1158/23,742	938/24,653	
3	310/7701	225/7858	32.4	31.9	4.0	2.9	p < 0.001	1.3	0.9	p < 0.001
			7701/23,742	7858/24,653	310/7701	225/7858		310/23,742	225/24,653	
4	415/1180	280/1175	5.0	4.8	35.2	23.8	p < 0.001	1.8	1.1	p < 0.001
			1180/23,742	1175/24,653	415/1180	280/1175		415/23,742	280/24,653	
5	1856/2304	1764/2535	9.7	10.3	80.6	65.6	p < 0.001	7.8	7.2	*p* = 0.0056
			2304/23,742	2535/24,653	1856/2304	1764/2535		1856/23,742	1764/24,653	
6	473/494	420/493	2.1	2.0	95.8	85.2	p < 0.001	2.0	1.7	*p* = 0.018
			494/23,742	493/24,653	473/494	420/493		473/23,742	420/24,653	
7	250/283	245/286	1.2	1.2	88.3	85.7	*p* = 0.34	1.1	1.0	*p* = 0.516
			283/23,742	286/24,653	250/283	245/286		250/23,742	245/24,653	
8	210/312	176/305	1.3	1.2	67.3	57.7	*p* = 0.014	0.9	0.7	*p* = 0.035
			312/23,742	305/24,653	210/312	176/305		210/23,742	176/24,653	
9	105/107	67/68	0.5	0.3	98.1	98.5	*p* = 0.84	0.4	0.3	*p* = 0.0016
			107/23,742	68/24,653	105/107	67/68		105/23,742	67/24,653	
10	313/1087	281/1048	4.6	4.3	28.8	26.8	*p* = 0.31	1.3	1.1	*p* = 0.075
			1087/23,742	1048/24,653	313/1087	281/1048		313/23,742	281/24,653	

The greatest 1.1% decrease was detected in 2 group with an absolute decrease in the overall CS from 4.9% to 3.8%. The greatest contribution to the overall CS rate was made by groups 1, 2 and 5 (previous CS, single cephalic, ≥ 37 weeks) (Table 2). These groups were identified as “target groups” and were responsible for 67.5% (4303/6379) of all CSs in 2012. The study analysis revealed that the CS rate decreased in 9 of 10 groups in 2014. A statistically significant decrease was detected not only in the “target groups”, but also in groups 3, 4, 6 and 8 (Table 2). In addition to this, the absolute contribution of all groups to the overall CS rate decreased.

Of particular importance though the combination of Groups 1 and 2 (the single cephalic nulliparous pregnancy at term) and Groups 3 and 4 (the single cephalic multiparous pregnancy without a scar at term) also showed a statistically significant reduction in CS rate (Tables 3 and 4). After comparison of nulliparous or multiparous with term single cephalic pregnancies and spontaneous or induced labours and prelabour cesarean sections in 2012 and 2014, we found a statistically significant decrease in the CS rate too (Tables 3 and 4).

**Table 3:** The number of CS in 1 and 2 groups and their absolute contribution to the overall CS rate in 2012 and 2014

	2012	2014	2012	2014	2012	2014		2012	2014	
Group	Number of CS over total number of women in 1 and 2 groups		Relative size of 1 and 2 groups (%)		CS rate in 1 and 2 groups (%)		p value	Absolute contribution to overall CS rate (%)		p value
1 and 2	2447/10,274	2147/10,885	43.3	44.2	23.8*	19.7*	p < 0.001	10.3*	8.7*	p < 0.001
			10,274/23,742	10,885/24,653	2447/10,274	2147/10,885		2447/23,742	2147/24,653	

*p < 0.05

**Table 4:** The number of CS in 3 and 4 groups and their absolute contribution to the overall CS rate in 2012 and 2014

	2012	2014	2012	2014	2012	2014		2012	2014	
Group	Number of CS over total number of women in 3 and 4 groups		Relative size of 3 and 4 groups (%)		CS rate in 3 and 4 groups (%)		p value	Absolute contribution to overall CS rate (%)		p value
3 and 4	725/8881	505/9033	37.4	36.6	8.2*	5.6*	p < 0.001	3.1*	2.0*	p < 0.001
			8881/23,742	9033/24,653	725/8881	505/9033		725/23,742	505/24,653	

*p < 0.05

Despite the statistically significant decrease in the CS rate in 2014, there was no statistically significant increase in overall perinatal mortality (Table 5) or in groups 1, 2 and 5, which made the greatest contribution to the overall CS rate (Table 6).

**Table 5:** Perinatal mortality in 2012 (total number of births 28,582) and 2014 (total number of births 28,200)

	2012	2014	p value
Antepartum deaths	92	111	0.1527
Intrapartum deaths	21	19	0.7871
Not defined	5	3	0.4902
Early neonatal deaths	31	33	0.7642
Perinatal mortality	149	166	0.2801

**Table 6:** The number of antepartum and intrapartum deaths in 2012 (total number of births 28,582) and 2014 (total number of births 28,200) in certain groups

Group	2012	2014		2012	2014	
	Number of antepartum deaths		p value	Number of intrapartum deaths		p value
1 and 2	18	14	0.5029	2	3	0.6455
3 and 4	13	18	0.3472	5	2	0.2627
5	2	1	0.5687	0	0	
Overall	33	33	0.9601	7	5	0.5823

*p < 0.05

## Discussion

We have interpreted our results following the recommendations provided by Robson et al. [8]. Groups 1, 2 and 5 normally account for two-thirds of the overall CS rate with group 5 as normally the largest contributor [8]. This was confirmed by our study and those groups were identified as “target groups”. The results showed that Lithuanian hospitals managed to reduce the CS rate not only in the “target groups”, but in almost all groups. The only group in which the CS rate increased was Group 9 and this was a direct consequence of improved data collection and subsequent classification. The number of women in group 9 decreased from 107 to 68 in 2014 although the CS rate (98.5%) in this group was still short of the 100% it should be. This shows that hospital staff still requires further training in correct classification. Although the “recommended” CS rate in group 1 is 10%, Lithuania’s hospitals did not manage to reach this rate in the course of two years, but decreased it from 16% (1289/8057) to 14% (1209/8646) [8]. Based on the ratio of group sizes between group 1 and group 2, the rate of inductions and pre-labour CSs in Lithuania was not high in 2012. Despite the fact, that the hospitals managed to reduce the CS rate in group 2, it still remained approximately 10% higher (41.9%) than the “recommended” rate (Table 2) [8].

Our results showed an increase in the number of women in group 5 in 2014. This may be attributed to the growing primary CS rate in Lithuania or possibly an improvement in data collection. According to national data, the CS rate in Lithuania nearly tripled, from 9.6% in 1995 to 26.0%, in 2012 [9]. However now by carrying out fewer CSs in nulliparous women, there will be fewer repeat CSs in the future. The CS rate in group 5 went down at the end of our study, from 80.6% (1856/2304) to 69.6% (1764/2535), but didn’t reach the “recommended” rate of 50–60% [8].

We have shown in our study that the Robson classification was useful not only for identifying the target groups of women who influence the CS rate the most, but seemed to work as an audit tool that significantly reduced the CS rate without any statistically significant change in the perinatal mortality. No specific clinical intervention in clinical practice was implemented during the study period. Moreover, no internal audit of any specific cases was performed. We analysed only the distribution of deliveries among Robson’s groups, according to the data presented by the individual hospital’s clinicians. These data were analysed and presented to health care policy-makers and hospital staff who implemented the Robson classification in their everyday clinical practice, and then subsequently discussed the results.

All the institutions involved in the whole study agreed that this should not be a “one-day action”, but the beginning of a new national strategy. Therefore, the Robson classification and staff feedback helped to reduce the CS rate. This whole study in its entirety had a lot in common with the original description of the Hawthorne effect, that when an individual or a group of people know that they are being observed, their behaviour changes and productivity increases [10].

A similar positive effect of the clinical audit cycle has been reported by other researchers as well. Saha et al. reported a 10.6% decrease of the overall CS rate at the Obstetrics and Gynecology Department of the Calcutta National Medical College and Hospital [11]. The decrease from 29% to 18.4% was observed after a new delivery management protocol was implemented and a new classification system was introduced in clinical practice [11]. A 10.3% (from 36.8% to 26.5%) and a 2.5% reduction in the overall CS rate was reported at the Van Buren Hospital in Chile and at Pembury Hospital in the United Kingdom respectively, after the Robson classification was introduced [4, 12]. The Haga Hospital in the Netherlands introduced a cesarean section audit and during daily report meetings all the CS’s and their indications were discussed [13]. A 4.7% decrease (from 23.4% to 18.7%) was achieved [13]. However, a study performed by Abdel-Aleem et al. has shown a 6% (from 32% to 38%) increase in the CS rate after the audit performed at Assiut University in Egypt [14]. According to the authors, this might have been due to the short duration (two months) of that study.

As far as we are aware there are no published data on the impact of a national implementation of Robson’s classification leading to an effect on the national CS rate. The strength of our study is that we have included the vast majority of Lithuania‘s hospitals and analysed more than 80% of deliveries in Lithuania. Therefore, our data may be treated as representative of the whole country. Moreover, with the achievement of a decreased CS rate without a negative impact on the perinatal outcomes in our country, this classification has now become compulsory in all Lithuanian hospitals by ministerial order as of 1 January 2016.

A possible limitation of our study is that the deliveries were not classified by an expert in the Robson classification. Hospital clinicians carried out the classification of deliveries and it was their first experience in this field. Perinatal data were recorded by the Institute of Hygiene Health Information Centre of Lithuania. No independent audit was performed to verify that the deliveries had been classified correctly or the perinatal mortality related data were correct. Moreover, we did not analyse any other data related to pregnancy, delivery and fetal or qualitative feedback from either the woman or the staff. Therefore, we are going to continue our project focusing on those factors mentioned above and to try and improve our national perinatal audit going forward in the future.

## Conclusion

The Robson classification can work as an audit tool to identify the groups that have the greatest impact on the CS rate. It also helps to develop a strategy focussing on the reduction of the CS rate.

## Abbreviations

CS: caesarean section
LSOG: Lithuanian Society of Obstetricians and Gynecologists
LUHS: Lithuanian University of Health Sciences
